# A simple calculation formula for the insertion depth of catheter of the central venous access port in Chinese patients

**DOI:** 10.1016/j.heliyon.2024.e40328

**Published:** 2024-11-12

**Authors:** Shan Chen, Hanbin Xie, Ren Wang, Weiqiang Chen, Jun Liu, Jibin Xing, Shangrong Li

**Affiliations:** Department of Anesthesiology, The Third Affiliated Hospital, Sun Yat-sen University, Guangzhou City, 510630, Guangdong Province, China

**Keywords:** Totally implantable venous access ports (TIVAPs), The depth of insertion of venous catheter, Simple calculation formula

## Abstract

**Background:**

The catheter tip placed between the T6 and T7 vertebrae is recognized as an optimal position for the totally implantable venous access ports (TIVAPs). This study aimed to propose a simple formula for calculating the optimal insertion depth of the right internal jugular central venous catheter (CVC) of TIVAP in Chinese patients.

**Methods:**

This was a prospective observational study. The medical records of 151 patients with TIVAPs placement were retrospectively analyzed. Patient's height and actual insertion depth of CVC were used to build a simple linear regression model. The Peres' formula was revised to better calculate the optimal insertion depth of the right internal jugular CVC of TIVAPs, accounting for population-specific anatomical variations observed in Chinese patients. A total of 158 patients scheduled for TIVAPs placement were prospectively recruited. The insertion depth of catheter was preoperatively determined according to the established formula, and postoperative chest X-ray was performed to determine the position of catheter tip. The rate of proper position of catheter (the catheter tip within T6 or T7 vertebrae) was calculated to assess the clinical feasibility and effectiveness of the new formula.

**Results:**

The proper position rate was 64.24 % (97/151) in the retrospective group. According to patients' height and insertion depth of CVC of these 97 cases with proper position, Peres' formula was revised to ‘insertion depth = 0.1∗height – 4 (cm)’. Based on insertion depth predicted by the new formula, the proper position rate in the prospective group reached 86.08 % (136/158).

**Conclusion:**

The simple formula for calculating the insertion depth of right internal jugular CVC of TIVAPs is clinically feasible for Chinese patients.

## 1 Background

Totally implantable venous access port (TIVAP) is a closed venous infusion system that is completely buried under the skin for a long time, which consists of a catheter system placed in the central vein and a piercing injection port buried under the skin [1]. The TIVAP is widely used for the infusion of chemotherapeutic drugs, parenteral nutrition support, and fluid supplements [2]. Nevertheless, central venous catheterization has been reported to be associated with various complications, such as pneumothorax, hemothorax, arterial puncture, infection, arrhythmia, cardiac tamponade, and thrombosis [[3], [4], [5]]. Proper position of the central venous catheter (CVC) tip can reduce the incidence of associated complications [[6], [7], [8]]. Growing evidence suggests that a catheter tip positioned at the junction of the superior vena cava and the right atrium (located at the T6-T7 vertebrae on chest radiograph) allows for safer and more effective TIVAP use [[9], [10], [11]]. The position of the catheter tip is determined by the insertion depth of CVC.

Unlike other CVC placements, the insertion of a CVC for TIVAP involves positioning the catheter in a completely subcutaneous system, which requires precise depth calculation to avoid both under-insertion, which could result in catheter misplacement, and over-insertion, which could lead to complications such as cardiac tamponade. Additionally, the extended duration of TIVAP placement under the skin adds further complexity, making correct tip placement even more critical for long-term use. These procedural differences highlight the importance of optimizing the insertion depth specific to TIVAPs to minimize complications and ensure effective function over time.

To optimize CVC position, in 1990, Peres has proposed a formula to preoperatively predict the insertion depth of the right internal jugular CVC (height/10, cm) [12], which is simple and widely used in the clinic. It has been shown that based on the Peres' formula, the proper position rate of CVC tip ranges between 48% and 74 % [[13], [14], [15]]. However, the Peres’ formula may not be appropriate for directly used to calculate the insertion depth of CVC of TIVAP in Chinese patients since it is established based on Western populations.

Therefore, this study aimed to explore whether adjustments to the Peres formula could improve the accuracy of CVC placement for TIVAP in Chinese patients and propose a simple formula for calculating the optimal insertion depth of the right internal jugular CVC for this specific population, considering the unique factors associated with TIVAP.

## 2 Results

### 2.1 Demographic and clinical characteristics

A total of 309 patients were included in this study, including 151 cases in the retrospective group and 158 cases in the prospective group. As shown in Table 1, the mean age was 51.27 ± 9.88 years, the gender ratio was 1:7.58 (male/female = 36/273). The mean patient's height was 158.50 ± 6.13 cm, and the insertion depth of CVC was 11.88 ± 0.67 cm. There was no significant difference in characteristics between the retrospective and prospective groups (all P > 0.05).

**Table 1:** Clinical characteristic of all included patients.

Parameters	Retrospective group (n = 151)	Prospective group (n = 158)	All (n = 309)	P
Sex				0.393
Male	20 (13.25 %)	16 (10.13 %)	36 (11.65 %)	
Female	131 (86.75 %)	142 (89.87 %)	273 (88.35 %)	
Age, year	50.99 ± 10.07	51.53 ± 9.71	51.27 ± 9.88	0.629
Height, cm	158.14 ± 5.79	158.84 ± 6.43	158.50 ± 6.13	0.314
Insertion depth of CVC, cm	11.84 ± 0.70	11.91 ± 0.64	11.88 ± 0.67	0.426

### 2.2 Retrospective group and peres estimation

**Fig. 1:**
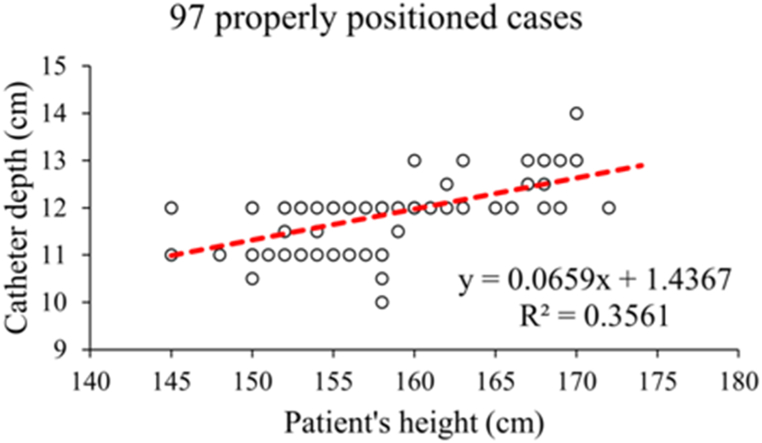
The scatter plot and regression line between patient's height and insertion depth of CVC of the 97 cases with a proper position of catheter.

In the retrospective group, the patient's insertion depth of CVC was determined based on surgeons' clinical experience. The estimated insertion depth of CVC was calculated according to Peres' formula (insertion depth = 0.1∗height) for comparing with the actual insertion depth. It was found that 64.24 % (97/151) patients in the retrospective group had the catheter tip within T6 or T7 vertebrae (defined as “proper position of catheter”). Patient's height and actual insertion depth of CVC of these 97 cases were used to build a simple linear regression model (Fig. 1), and the regression formula was ‘insertion depth = 0.0659∗height +1.4367’.

This regression formula was also used to estimate the depth in the 54 patients without proper position of catheter. As shown in Table 2, there was no significant difference in demographic and clinical characteristics between the patients with or without proper position of catheter (all P > 0.05) in the retrospective group. Table 3 showed the distribution of catheter position of all cases in the retrospective group.

**Table 2:** Comparison between patients with or without proper position of catheter based on Peres estimation in the retrospective group.

Parameters	aProper position of catheter		All (n = 151)	P
	No (n = 54)	Yes (n = 97)		
Sex				0.281
Male	5 (9.26 %)	15 (15.46 %)	20 (13.25 %)	
Female	49 (90.74 %)	82 (84.54 %)	131 (86.75 %)	
Age, year	49.06 ± 9.69	52.04 ± 10.17	50.99 ± 10.07	0.083
Height, cm	158.37 ± 5.22	158.01 ± 6.11	158.14 ± 5.79	0.716
Insertion depth of CVC, cm	11.84 ± 0.75	11.85 ± 0.67	11.84 ± 0.70	0.982
Peres results	15.84 ± 0.52	15.80 ± 0.61	15.81 ± 0.58	0.716
bPeres residuals	3.99 ± 0.69	3.96 ± 0.58	3.97 ± 0.62	0.714
Regression results	11.87 ± 0.34	11.85 ± 0.40	11.86 ± 0.38	0.716

a Proper position of catheter was defined as placement of the catheter tip between T6 or T7 vertebrae.

b Peres residual was defined as the difference between the actual insertion depth of CVC and the Peres estimation.

**Table 3:** Catheter position in the retrospective group.

Parameters	aProper position of catheter		All (n = 151)	P
	No (n = 54)	Yes (n = 97)		
Catheter position				<0.001
T3	1 (1.85 %)	0	1 (0.66 %)	
T4	8 (14.81 %)	0	8 (5.30 %)	
T5	39 (72.22 %)	0	39 (25.83 %)	
T6	0	63 (64.95 %)	63 (41.72 %)	
T7	0	34 (35.05 %)	34 (22.52 %)	
T8	5 (9.26 %)	0	5 (3.31 %)	
T9	1 (1.85 %)	0	1 (0.66 %)	

a Proper position of catheter was defined as placement of the catheter tip between T6 or T7 vertebrae.

### 2.3 New formula revised from peres estimation

**Fig. 2:**
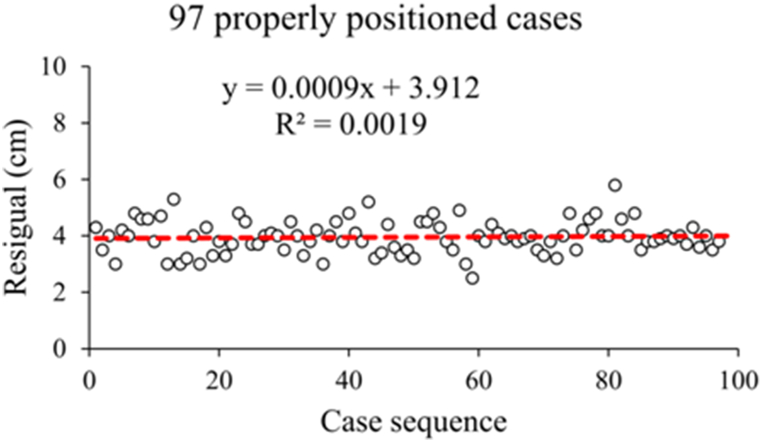
The residuals (depth estimated by Peres' formula subtracted actual insertion depth of CVC) scatter plot and regression line.

The regression formula from the 97 properly positioned patients was too complicated and sample restricted. Therefore, a new formula was brought up through the observation of Peres residuals (Fig. 2). It was found that after a simple ratio transformation (depth = 0.1∗height), there was still a mean difference of 3.96 ± 0.58 cm (median 4 cm) between the Peres estimation and the actual insertion depth of CVC. Thus, the revised new formula ‘depth = 0.1∗height - 4’ was proposed. The estimated positioning depth by the new formula within the 97 patients was 11.80 ± 0.61 cm, which was not significantly different from the actual insertion depth of CVC (P = 0.453).

### 2.4 Prospective group and new formula estimation

The new formula estimation was adopted to preoperatively determine the insertion depth of CVC the in prospective group. According to the postoperative anteroposterior chest X-ray, 86.08 % (136/158) cases had proper CVC position (Table 4). There was no significant difference in demographic and clinical characteristics between the patients with or without proper position of catheter (all P > 0.05) in the prospective group. Table 5 showed the distribution of catheter position of all cases in the prospective group.

**Table 4:** Comparison between patients with or without proper position of catheter based on the new formula in the retrospective group in the prospective group.

Parameters	aProper position of catheter		All (n = 158)	P
	No (n = 22)	Yes (n = 136)		
Sex				0.701
Male	1 (4.55 %)	15 (11.03 %)	16 (10.13 %)	
Female	21 (95.45 %)	121 (88.97 %)	142 (89.87 %)	
Age, year	54.50 ± 10.33	51.05 ± 9.56	51.53 ± 9.71	0.123
Height, cm	156.59 ± 5.79	159.21 ± 6.47	158.84 ± 6.43	0.077
Insertion depth of CVC, cm	11.71 ± 0.60	11.94 ± 0.64	11.91 ± 0.64	0.129
Peres results	15.66 ± 0.58	15.92 ± 0.65	15.88 ± 0.64	0.077
bPeres residuals	3.95 ± 0.26	3.98 ± 0.22	3.98 ± 0.22	0.445
Regression results	11.76 ± 0.38	11.93 ± 0.43	11.90 ± 0.42	0.077
New formula result	11.66 ± 0.58	11.92 ± 0.65	11.88 ± 0.64	0.077

a Proper position of catheter was defined as placement of the catheter tip between T6 or T7 vertebrae.

b Peres residual was defined as the difference between the actual insertion depth of CVC and the Peres estimation.

**Table 5:** Catheter position in the prospective group.

Parameters	aProper position of catheter		All (n = 158)	P
	No (n = 22)	Yes (n = 136)		
Catheter position				<0.001
T4	2 (9.09 %)	0	2 (1.27 %)	
T5	6 (27.27 %)	0	6 (3.80 %)	
T6	0	74 (54.41 %)	74 (46.84 %)	
T7	0	62 (45.59 %)	62 (39.24 %)	
T8	13 (59.09 %)	0	13 (8.23 %)	
T9	1 (4.55 %)	0	1 (0.63 %)	

a Proper position of catheter was defined as placement of the catheter tip between T6 or T7 vertebrae.

### 2.5 Comparisons between retrospective and prospective groups

The proper position rate was significantly higher in the prospective group than in the retrospective group (Table 6, 86.08 % *vs*. 64.24 % P < 0.001). The detailed distribution of catheter position was also significantly different between the two groups (Table 6, P < 0.001). There were more patients with T6/T7 positions in the prospective group.

**Table 6:** Proper position rate between retrospective and prospective group.

Parameters	Retrospective group (n = 151)	Prospective group (n = 158)	All (n = 309)	P
aProper position of catheter				<0.001
No	54 (35.76 %)	22 (13.92 %)	76 (24.60 %)	
Yes	97 (64.24 %)	136 (86.08 %)	233 (75.40 %)	
Catheter position				<0.001
T3	1 (0.66 %)	0	1 (0.32 %)	
T4	8 (5.30 %)	2 (1.27 %)	10 (3.24 %)	
T5	39 (25.83 %)	6 (3.80 %)	45 (14.56 %)	
T6	63 (41.72 %)	74 (46.84 %)	137 (44.34 %)	
T7	34 (22.52 %)	62 (39.24 %)	96 (31.07 %)	
T8	5 (3.31 %)	13 (8.23 %)	18 (5.83 %)	
T9	1 (0.66 %)	1 (0.63 %)	2 (0.65 %)	

a Proper position of catheter was defined as placement of the catheter tip between T6 or T7 vertebrae.

**Fig. 3:**
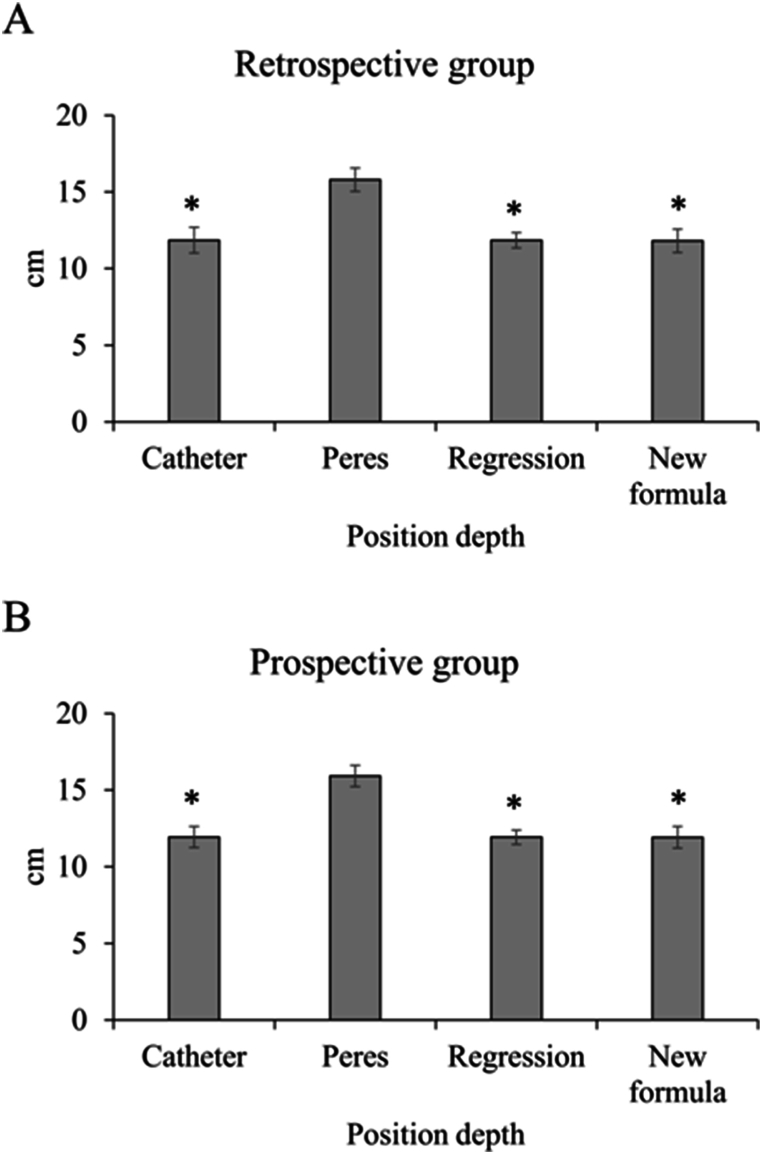
The position depth, including actual catheter, estimation by Peres, regression, and new formula. There were retrospective group (A) and prospective group results (B). ∗ means P < 0.05 compared with Peres estimation.

The insertion depth of CVC was compared among the actual insertion depth of CVC, Peres’ estimation, regression estimation, and estimation by the new formula. In both the retrospective group (Fig. 3A) and prospective group (Fig. 3B), the result from Peres estimation was significantly higher than the other 3 subgroups (all P < 0.05). However, there was no significant difference among the results from the actual insertion depth of CVC, regression estimation, and estimation by the new formula (all P > 0.05).

## 3 Discussion

Peres' formula is proposed based on the body of Western populations [12], which are generally taller than the Chinese population. This difference can lead to error in the proportion of body shape and body surface positioning. In addition, Peres’ formula is designed for central venous catheterization, in which the insertion depth of the catheter is determined based on the catheter scale outside the skin. In the TIVAP, however, the catheter is completely buried under the skin, and the depth of catheter placement is based on the scale corresponding to the plane of the transverse process of the C6 vertebra. Given these differences, we explored whether adjustments to the Peres formula would improve the accuracy of CVC placement for TIVAP. Our study identified certain deviations from the predicted values, which could potentially be associated with the unique subcutaneous nature of TIVAP and its prolonged placement. However, further analysis is needed to determine the exact cause of these deviations. This preliminary observation suggests that adjustments to the insertion depth formula might be necessary when applying it to TIVAPs, though additional investigation is required to confirm this.

In our study, we did not initially reference the Peres formula when inserting the infusion ports. Instead, we utilized a formula proposed by Duan et al., in 2012 [16], which was specifically developed for the adult population in Southwestern China. This formula for right-sided internal jugular central venous catheter insertion depth is calculated as follows: insertion depth (cm) = height (cm) × 0.06 + 4. Using this formula, we inserted infusion ports for 50 patients between 2015 and 2016. However, post-operative X-rays showed a catheter tip accuracy rate of only 40 % (unpublished data). After evaluating the results, we concluded that the depth of the catheter measured as the exposed part of the catheter (from the puncture point to the tip) differed from the depth needed for infusion ports, where the catheter forms an "∩" shape through the subcutaneous tunnel to the port pocket. Hence, the two procedures require distinct depth calculations.

In this study, we aimed to establish a simple formula for calculating the optimal insertion depth of the right internal jugular central venous catheter of TIVAP in Chinese patients. In the retrospective analysis, among 151 patients with TIVAP placement, 97 patients had the proper position of catheter tip (T6 to T7 vertebrae), with a proper position rate of 64.24 % (97/151). Patient's height and actual insertion depth of CVC of these 97 cases with proper position were used to build a simple linear regression formula ‘insertion depth = 0.0659∗height +1.4367’. However, this formula is a bit complicated for clinical use.

Based on Peres' formula, the estimated mean catheter insertion depth of the 97 patients was 15.80 ± 0.61 cm, while the actual catheter placement depth was 11.84 ± 0.70 cm. The difference between the actual insertion depth of CVC and the Peres estimation (defined as Peres residuals) of the 97 patients exhibited normal distribution (P = 0.078), and the mean value was 3.96 ± 0.58 cm. Based on the scatter plot, the Peres residual is approximately equal to a constant of 3.912. Therefore, the Peres' formula was revised and simplified to ‘insertion depth = 0.1 × height– 4(cm)’. The Mann-Whitney test demonstrated that there was no difference in the distribution of CVC insertion depth calculated according to the regression equation and the revised Peres' formula (P = 0.331 > 0.05). Therefore, for convenience in clinical use, the revised Peres' formula was chosen as the simple calculation formula for the optimal insertion depth of the right internal jugular central venous catheter of TIVAP in Chinese patients.

To further validate the clinical feasibility of this new formula, 158 patients scheduled to receive TIVAPs placement via the right internal jugular vein were enrolled. Based on the preoperative estimation by the new formula, the proper position rate in these 158 cases reached 86.08 % (136/158), which was significantly higher than the proper position rate in the retrospective group. This result suggested that the preoperative estimation by the new formula can significantly improve the proper position rate of the catheter tip of TIVAPs, and this new formula is appropriate for clinical use.

Different from intraoperative real-time guiding catheterization methods, such as real-time ultrasound guidance [17,18], intracavitary electrocardiography-guided localization [19,20], and echocardiography [21,22], the formula method cannot avoid the occurrence of catheter ectopic. In this prospective study, 1 case (1/158, 0.63 %) of the catheter was ectopic into the right subclavian vein. The catheter was immediately reinserted to the superior vena cava under the guidance of intraoperative X-ray, and it could be used normally.

It has been reported that ultrasound-guided catheterization through the right internal jugular vein has a lower incidence of catheter ectopic than catheterization through anatomical landmarks [23] or catheterization through the left internal jugular vein [24]. However, once catheter ectopic is found in the postoperative chest X-ray, re-operation is needed for adjustment.

The study relies on bony landmarks for both the insertion point (C6) and the tip location (T6-T7). The use of soft tissue landmarks, such as the junction of the superior vena cava (SVC) and the right atrium, is commonly recommended for optimal catheter tip placement. However, techniques such as intraoperative real-time fluoroscopy, intracardiac electrocardiography, and transthoracic or transesophageal echocardiography, while highly accurate, often require specialized equipment and expertise that may limit their widespread application in routine clinical practice. Consequently, relying on bony landmarks remains a practical and reliable alternative in many settings. We recognize the anatomical variability in patients and the fact that bony landmarks may not correlate directly with vascular structures, which can influence the optimal depth of insertion. C6 was selected as the insertion point because it is situated at a level where the common carotid artery is relatively superficial and easily palpable. The internal jugular vein runs parallel to the common carotid artery, providing a stable reference point that reduces the risk of injuring adjacent structures, such as the pleura or thoracic duct. This choice is particularly beneficial in healthcare facilities lacking ultrasound guidance, allowing for precise catheter placement. Additionally, a study by Song et al. [11] involving 524 adult patients demonstrated that the junction of the SVC and right atrium can be reliably estimated to be approximately 2.4 vertebral units below the carina, corresponding to the T6-T7 level. This finding supports the continued use of postoperative chest X-rays to confirm catheter tip placement, striking a practical balance between accuracy and clinical feasibility. While our study focused on bony landmarks for their practicality, we acknowledge that the use of soft tissue landmarks is a valid consideration, particularly in patients with significant anatomical variability.

Although our study did not observe any complications due to the small sample size, literature suggests that correct catheter positioning is crucial. A retrospective study by Caers et al. [6] confirmed that catheter tip positioning significantly impacts the incidence of complications, particularly thrombosis. Similarly, Luciani et al. [25] highlighted that correct positioning at the junction of the superior vena cava and right atrium can lead to a significant reduction in catheter-related thrombosis rates.

While our study presents a revised formula for estimating catheter insertion depth in Chinese patients, several limitations must be acknowledged. Firstly, further research is necessary to confirm the effectiveness of the adjustments made to the Peres formula when applied to TIVAPs, as it was originally developed for CVCs. The observed deviations between the actual and estimated insertion depths may be partially attributable to differences in device characteristics and anatomical considerations unique to TIVAPs. This suggests that the adjustments we propose might not be universally applicable across different types of central venous devices. Additionally, our findings may not be generalizable beyond the specific population studied (Chinese patients). Although our analysis showed no significant effect of patient height on the accuracy of catheter placement, further validation in populations with broader height distributions is necessary. Future studies should focus on validating this new formula across diverse populations and clinical settings while exploring how variations in device design and application may impact the accuracy of the formula. Such investigations would enhance the understanding of its applicability and effectiveness in broader contexts.

## 4 Conclusions

In summary, the new formula established for calculating the insertion depth of right internal jugular CVC of TIVAPs is clinically feasible for Chinese patients. Compared with other intraoperative guidance catheter placement methods, the formula method to calculate the insertion depth of catheter of TIVAPs has the advantages of simple operation, no need for special equipment, and no increase in operating costs. Therefore, for hospitals without intraoperative guidance equipment, the new formula can be used to predict the optimal insertion depth of the right internal jugular central venous catheter of TIVAP in Chinese patients. For hospitals with intraoperative real-time guidance equipment, this formula can also be used as a reference to predict the depth of catheter insertion.

## 5 Methods

### 5.1 Study subjects

This was a prospective observational study. From March 2016 to April 2017, 225 adult patients with TIVAPs placement in the Department of Anesthesiology of the Third Affiliated Hospital of Sun Yat-sen University were retrospectively reviewed. Forty-one patients with TIVAPs placement not via the right internal jugular vein (via the right subclavian vein in 17 cases, via the left internal jugular vein in 20 cases, and via the left subclavian vein in 4 cases) were excluded. Thirty-three patients with missing data on height or catheter insertion depth were excluded, and 151 patients were finally included in the study.

To validate the clinical feasibility of the new formula, 158 patients who required placement of TIVAPs in the Department of Anesthesiology for treatment at the Third Affiliated Hospital of Sun Yat-Sen University from April 2018 to September 2019, were recruited. Patients aged ≥18 years old were included. Exclusion criteria: (1) with a history of cardiac large vessel variation, superior vena cava obstruction, bilateral lung or mediastinal disease; (2) with severe arrhythmia; (3) with a history of thrombosis or abnormal coagulation function; (4) The right internal jugular vein puncture site and the right subclavian burial site had a history of infection, injury, and radiation therapy; (5) with right breast cancer who still need subsequent surgery. Elimination case criteria: (1) with anatomical variation at the right puncture site; (2) severe adverse events during TIVAP placement leading to the abandonment of the operation; (3) persistent arrhythmia during TIVAP placement leading to unable to insert the catheter to the depth according to the formula estimation.

This study was conducted in accordance with the Declaration of Helsinki and approved by the institutional review board of the Third Affiliated Hospital, Sun Yat-sen University (No. [2018]02-013-01). Written informed consent was obtained from each patient.

### 5.2 Sample size calculation

It has been shown that based on the Peres’ formula, the proper position rate of CVC tip ranges between 48% and 74 % [[13], [14], [15]]. A sample size algorithm based on the exact mid-P method of confidence interval estimation was used to determine the sample size [26]. According to the pre-experiment results, it is expected that 85 % of the cases would have a proper position of catheter tip (between the T6 and T7 vertebrae). The target value of the proper position rate was 74 %, the test level was 0.025 on one side, and the test power was 90 %. At least 144 subjects were needed. If the dropout rate was 10 %, 159 subjects were required.

### 5.3 TIVAPs placement procedure

An implantable drug delivery device (B. Braun Medical Inc., USA) was adopted for TIVAP placement. The patient was placed in a supine position with a pillow on the right shoulder, and the head was tilted back 15° and slightly to the left. The mask was inhaled with 5 L/min of oxygen, and non-invasive blood pressure, ECG monitoring, and pulse oxygen saturation were monitored. B-ultrasound was used to observe the right internal jugular vein to confirm no structural variation, no deformity, no stenosis, no thrombus, no lymph node metastasis around the blood vessel. The puncture point was the plane of the transverse process of the C6 vertebra).

After local anesthesia, the catheter was inserted through the right internal jugular vein under the guidance of ultrasound, and the subcutaneous injection port was separated 1–2 cm below the right clavicle. A subcutaneous tunnel was established and the catheter was introduced to the injection port. The scale of the catheter in the neck incision was determined based on the new formula (0.1 × height (cm)-4), which is the insertion depth of the catheter. After the venous blood was withdrawn and normal saline was injected to confirm the patency, the incision was sutured and pressure bandaged. An anterior chest radiograph was performed immediately after surgery to confirm the position of the catheter tip.

### 5.4 Data collection

Main outcome measures: The position of the catheter tip on postoperative anteroposterior chest X-ray, and the catheter tip located between the T6 and T7 vertebrae was defined as a proper position of the catheter tip. Secondary outcome measures: General information: patient's age, height, weight, disease history, surgical history, medication history; intraoperative and postoperative complications related to TIVAP placement. Intraoperative complications included puncture failure, hemopneumothorax, arrhythmia, air embolism, local hematoma. Postoperative complications included catheter ectopic, local or systemic infection, inversion or displacement of the injection seat, catheter obstruction or displacement, catheter-related thrombosis, rupture or dislodgement of the catheter.

### 5.5 Statistical analysis

Continuous data were indicated using mean ± standard deviation (SD). For the comparisons between two groups, the student's independent *t*-test or Mann-Whitney *U* test (if normality was not assumed) were used. Categorical data were indicated with number and percentage (%), and the distribution would be tested with the Chis-square test or Fisher's exact test (if any expected value ≤ 5 was observed). Simple linear regression was used to estimate the association between two continuous variables (e.g., height and insertion depth of CVC). For the comparisons among position depth (actual catheter, estimated by Peres, regression, and by new formula) results, one-way repeated ANOVA and Fisher's LSD test as post-hoc comparisons were used. A P < 0.05 would be recognized as reaching the significance of each test, two-tailed. All analyses were performed using IBM SPSS Version 25 (SPSS Statistics V25, IBM Corporation, Somers, New York).

## CRediT authorship contribution statement

**Shan Chen:** Writing – review & editing, Writing – original draft, Validation, Formal analysis, Data curation. **Hanbin Xie:** Writing – review & editing, Writing – original draft, Formal analysis, Data curation. **Ren Wang:** Methodology, Formal analysis. **Weiqiang Chen:** Resources, Formal analysis. **Jun Liu:** Software, Methodology. **Jibin Xing:** Data curation. **Shangrong Li:** Supervision, Project administration, Conceptualization.

## Funding sources

This research did not receive any specific grant from funding agencies in the public, commercial, or not-for-profit sectors.

## Declaration of competing interest

The authors declare that they have no known competing financial interests or personal relationships that could have appeared to influence the work reported in this paper.
